# Randomised controlled trial of an innovative hypoglycaemia pathway for self-care at home and admission avoidance: a partnership approach with a regional ambulance trust

**DOI:** 10.29045/14784726.2022.03.6.4.3

**Published:** 2022-03-01

**Authors:** Andrew Willis, Helen Dallosso, Laura Gray, June James, Cat Taylor, Melanie Davies, Debbie Shaw, Niroshan Siriwardena, Kamlesh Khunti

**Affiliations:** University of Leicester; NIHR Applied Research Collaboration East Midlands (ARC-EM) ORCID iD: https://orcid.org/0000-0002-9671-2162; University Hospitals of Leicester NHS Trust; NIHR Applied Research Collaboration East Midlands (ARC-EM) ORCID iD: https://orcid.org/0000-0002-6732-0864; University of Leicester ORCID iD: https://orcid.org/0000-0002-9284-9321; University Hospitals of Leicester NHS Trust; University of Leicester; University Hospitals of Leicester NHS Trust; NIHR Leicester Biomedical Research Centre ORCID iD: https://orcid.org/0000-0002-9987-9371; East Midlands Ambulance Service NHS Trust; East Midlands Ambulance Service NHS Trust; University of Lincoln ORCID iD: https://orcid.org/0000-0003-2484-8201; University of Leicester; NIHR Applied Research Collaboration East Midlands (ARC-EM) ORCID iD: https://orcid.org/0000-0003-2343-7099

**Keywords:** diabetes management, hypoglycaemia, pre-hospital research

## Abstract

**Background:**

Hypoglycaemia is a common and potentially life-threatening condition in people with diabetes, commonly caused by medications such as insulin. Hypoglycaemic events often require in-patient treatment and/or follow-up with a diabetes specialist nurse (DSN) or GP to make adjustments to medication. This referral pathway commonly relies on patient self-referral to primary care, and as a result many patients are not actively followed up and go on to experience repeat hypoglycaemic events.

**Methods:**

Randomised controlled trial in partnership with East Midlands Ambulance Service NHS Trust. People with diabetes calling out an ambulance for a severe hypoglycaemic episode and meeting the eligibility criteria were randomised to either a novel DSN-led pathway or to their general practice for routine follow-up. Primary outcome was proportion of participants with a documented consultation with a healthcare professional to discuss the management of their diabetes within 28 days of call-out.

**Results:**

162 people were randomised to one of the pathways (73 DSN arm, 89 GP arm) with 81 (50%, 35 DSN, 46 GP) providing full consent to be followed up. Due to lower than anticipated randomisation and consent rates, the recruitment target was not met. In the 81 participants who provided full consent, there were higher rates of consultation following the call-out when referred to a DSN compared to primary care (90% vs. 65%). Of the 81 participants, 26 (32%) had a second call-out within 12 months.

**Conclusions:**

Consultation rates following the call-out were high in the DSN-led arm, but there was insufficient power to complete the planned comparative analysis. The study highlighted the difficulty in recruitment and delivery of research in pre-hospital emergency care. Further work is needed to provide more feasible study designs and consent procedures balancing demands on ambulance staff time with the need for robust well-designed evaluation of referral pathways.

## Introduction

Severe hypoglycaemia (SH) is a common side effect of medications taken to reduce blood glucose in people with type 1 and type 2 diabetes. It is defined as an episode of hypoglycaemia that the patient is unable to self-treat (American Diabetes Association, 2005) and is associated with increased morbidity and mortality (Elwen et al., 2015) and significantly lower quality of life. In the United Kingdom, ambulance trusts are the main provider of first-contact emergency medical services for SH. It is estimated that there are 70,000 to 100,000 emergency call-outs per year for hypoglycaemia at a cost of £13.6 million per year to the NHS, with each admission to hospital costing around £900 (Farmer et al., 2012).

Medications, including insulin and other oral hypoglycaemic agents, are most frequently associated with SH, but follow-up of people experiencing SH shows that in the United Kingdom, only 27–37% of patients contact their primary care services themselves to discuss medication changes or the cause of SH (Duncan & Fitzpatrick, 2016). Because of this, recurrent call-outs for SH occur within seven days in 2–7% of cases (Duncan & Fitzpatrick, 2016; Tsai et al., 2011), with this increasing over time to 10.6% within 14 days (Fitzpatrick, 2015), contributing to further healthcare costs and associated increased risk of mortality and morbidity.

In order to avoid recurrent call-outs, several ambulance trusts in the United Kingdom operate ‘treat and refer’ protocols for SH (Snooks et al., 2013), whereby the ambulance crew provide treatment to stabilise the patient at the scene of the incident, and only convey patients to hospital in more serious cases or where symptoms are ongoing. Data from the East Midlands show that the majority of people who experience SH remain at home following treatment from an ambulance clinician (Khunti et al., 2013), and best practice guidelines encourage referral to their primary care team or to their local diabetes care service, to enable a review of the patient and their management plan to be carried out (Joint British Diabetes Societies for Inpatient Care, 2013). Local specialist referral pathways have been implemented in various sites (Buchanan et al., 2014; Sampson et al., 2017), and reviews of the literature (Bell & Fitzpatrick, 2016; Bloomer, 2019; Collenette, 2018) suggest that specialist diabetes referrals by ambulance staff after an SH emergency may be beneficial to patients. However, the evidence is limited, with the majority of studies being service evaluations (James et al., 2013; Lerner et al., 2003; Walker et al., 2006) and non-randomised controlled trials.

The aim of this study was to evaluate a hypoglycaemia referral pathway which involves the patient having a telephone consultation with a diabetes specialist nurse (DSN) following a call-out for SH (James et al., 2013) compared to standard care, which in this case was referral by ambulance clinicians to the patient’s own GP. The pathway was developed by the Integrated Care Diabetes Service at University Hospitals of Leicester NHS Trust in collaboration with the emergency medical service leads from East Midlands Ambulance Service NHS Trust.

## Methods

The study was a two-centre randomised controlled trial (RCT) with two parallel arms and balanced randomisation (1:1). Participants randomised to the DSN arm were offered a telephone consultation with a locally based DSN, during which the DSN discussed current medications, suggested medication changes (where appropriate) and offered further advice on avoiding further SH episodes. Participants randomised to the GP arm had their details sent to their GP with a recommendation to contact the participant to discuss their diabetes management, in line with current best practice at the time of recruitment.

### Referral and randomisation

Ambulance staff working for East Midlands Ambulance Service NHS Trust and based in Lincolnshire or Northamptonshire were invited to complete training in the referral procedures. Trained staff were provided with packs of five sequentially numbered envelopes with instructions inside on which arm to randomise the patient to. The randomisation list was completed by an independent statistician using a block size of four. Due to the need to provide a simple procedure for ambulance staff to randomise, it was not possible to stratify randomisation by demographic characteristics.

### Eligibility for referral

**Figure 1:**
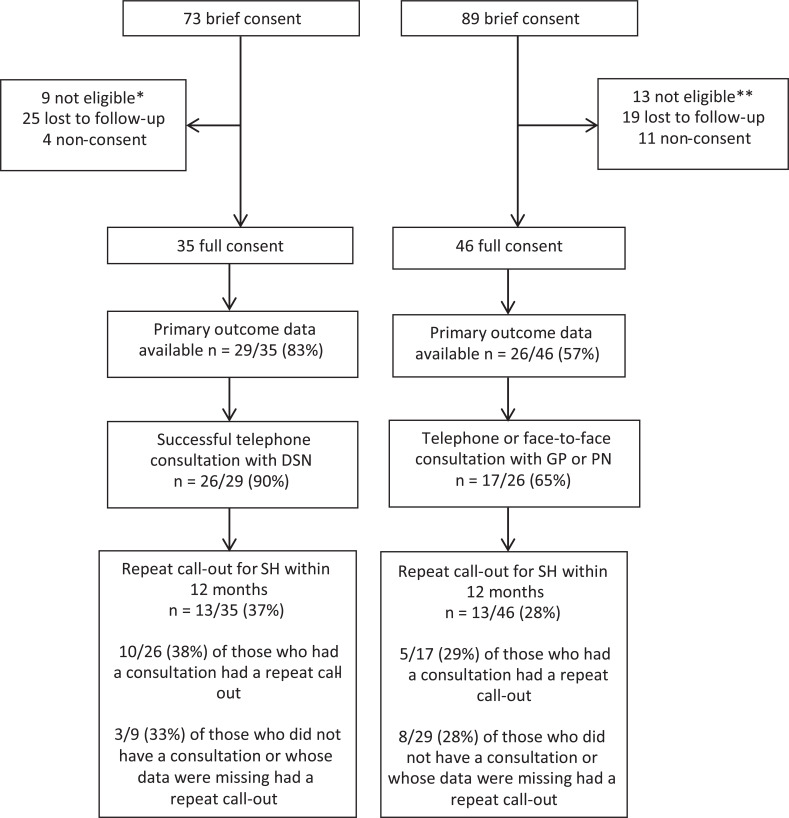
Figure 1. Flow of participants through the study.

Individuals were eligible for randomisation if they had diabetes, had called the ambulance service for an SH event (confirmed by attending ambulance staff on the basis of a capillary blood glucose level of < 4.0 mmol/L), were aged 18 years and over, were willing and able to give informed consent, were able to speak and read English, were living in and registered with a general practice in Lincolnshire or Northamptonshire and were responsible for their own care and/or medication (Table 1). For the patients conveyed to hospital, those who were not admitted or were admitted for less than 48 hours remained eligible. Those admitted to hospital for more than 48 hours were excluded, as current best practice ensures that these patients’ care pathway while in hospital already includes input from a DSN (Figure 1).

**Table 1.** Study referral criteria.

Inclusion criteria	Exclusion criteria*
(Type 1 or 2 diabetes and attended by service for ‘hypo’ (blood glucose < 4 mmol/L)	Not responsible for their own care and/or medication
Age ≥ 18 years	Lives in residential or care home
Able to speak and read English	Not registered with GP in Lincolnshire or Northamptonshire
Registered with GP in Lincolnshire or Northamptonshire	
Responsible for their own care and/or medication	
Willing and has capacity to give brief consent	

*Participants who were conveyed and admitted to hospital for > 48 hours were excluded from the study.

*5 admitted to hospital > 48 hours, 2 previously referred, 1 lived out of area, 1 no information.

**5 admitted to hospital > 48 hours, 3 lived out of area, 1 not registered with GP, 1 in nursing home, 1 not diabetic, 2 no information. DSN = diabetes specialist nurse; SH = severe hypoglycaemia.

### Consent procedures

As individuals were recovering from an SH event, there was a risk they lacked the cognitive capacity to provide informed consent and/or fully process and retain information pertaining to the study (Cain et al., 2003). The research ethics committee therefore requested that consent be taken in two stages. After treating the individual’s symptoms, the ambulance staff checked their eligibility and obtained ‘brief consent’ to be randomised to one of the two pathways. After ‘brief consent’ was taken, the ambulance staff opened a randomisation envelope and informed the participant that they would either be called by a DSN for a consultation about their diabetes and the SH event (DSN arm) or that the details of the call-out would be sent to their GP (GP arm) with a request to follow up. Participants in both arms were given an information booklet about the management of hypoglycaemia. Ambulance staff then transferred the information to the research team who referred the participant to the appropriate pathway. At this point the research team initiated the second stage of the consent process. Participants conveyed to hospital (or in some cases a member of their family) were contacted to find out whether they had been admitted to hospital. If they had been admitted to hospital for more than 48 hours they were withdrawn from the study. If they were not admitted or were admitted for less than 48 hours, they were sent a patient information leaflet and asked to provide ‘full consent’ to follow-up data being collected. Consent was provided by telephone or by returning a signed consent form. Participants not conveyed to hospital were sent a patient information leaflet and provided ‘full consent’ by telephone or by returning a signed consent form. Outcome data were collected on participants who provided full consent.

### Outcome data

The overall objective of the pathway was to reduce the chance of a further ambulance/emergency medical services call-out occurring, but a primary outcome of reduction in repeat call-out rate would have necessitated an unachievable sample size. The primary outcome was therefore documented evidence that the hypoglycaemic episode was discussed (within four weeks of the call-out) with a healthcare professional, and that relevant advice was given and/or changes made to prescribed medication. The four-week time frame was chosen to allow patients randomised to the GP arm to book and attend an appointment at their surgery, also allowing for any delayed/cancelled appointments.

Primary outcome data were either provided by the local diabetes care service (DSN arm) or the participant’s practice was approached for the information (GP arm). Information on whether the participants made a further call-out to the ambulance service for an SH event during the following 12 months was provided by East Midlands Ambulance Service NHS Trust from their central records. The trust also provided information on the total number of call-outs and repeat call-outs associated with SH that occurred over the region during the study period. Secondary outcome data including overall rates and cases with the chief complaint of diabetic problems were extracted directly from the Computer Aided Dispatch (CAD) system. The records were then linked with the corresponding records in the electronic and paper patient report form (PRF) systems and those with a clinical impression of hypoglycaemia on the PRF used as the comparison data.

### Sample size and statistical analysis

There were no reliable data on which to base a formal sample size calculation. Therefore, we assessed a range of plausible intervention differences based on obtaining primary outcome data for 150 participants (this number was deemed reasonable in the recruitment period). Assuming that full consent would not be obtained for 20% of participants and that primary outcome data would be unobtainable for 10%, around 216 randomisations were needed to obtain this sample size.

The percentage of participants fulfilling the primary outcome were to be reported by randomised group, with logistic regression used to assess the difference between the two groups in terms of the proportion satisfying the primary outcome. Sensitivity analyses were to be carried out on an intention-to-treat basis (using multiple imputation to replace missing outcome data) and a per-protocol basis (excluding participants not receiving their randomised intervention).

## Results

In total, 165 ambulance staff completed training in the randomisation and consent procedures, which amounted to approximately 25% of ambulance staff working in the two recruitment sites. Randomisation took place between 29 April 2016 and 30 June 2018 (26 months), during which time 162 people provided brief consent and were randomised to one of the two pathways (Figure 1). The number providing brief consent in the DSN arm was slightly lower than in the GP arm (73 and 89 respectively) but both groups were well matched (Table 2). Full consent was subsequently obtained from 81 participants (50% of those who provided brief consent). At this point, it was agreed the recruitment target would not be reached and referral was stopped.

**Table 2.** Demographics of 162 participants providing brief consent and 81 participants providing full consent.

Participants providing brief consent (n = 162)			
	DSN arm (n = 73)	GP arm (n = 89)	Total (n = 162)
Age (y), med (IQR)	65 (45–75)	68 (53–68)	67 (48–75)
Male N (%)	38 (52.1%)	54 (60.7%)	92 (56.8%)
Full consent N (%)	35 (48%)	46 (52%)	81 (50%)
Participants providing full consent (n = 81)			
	DSN arm (n = 35)	GP arm (n = 46)	Total (n = 81)
Age (y), med (IQR)	65 (53–72)	69 (56–75)	67 (54–730
Male N (%)	17 (48.6%)	24 (52.2%)	41 (50.6%)
Type 1 diabetes, N* (%)	16/32 (50%)	16/32 (50%)	32/64 (50%)
Insulin only, N (%)	21/25 (84%)	10/13 (76.9%)	31/38 (81.6%)

*‘Diabetes type’ data were available for 64 out of 81 participants.

DSN = diabetes specialist nurse; IQR = interquartile range.

Table 2 provides demographic data on the 162 referrals – the 81 who did not provide full consent and the 81 who did provide full consent. The GP and DSN arms were matched in terms of age and sex, but there were small differences between those who did and did not provide full consent. General practices were approached for clinical data (type of diabetes, medication, etc.) and for information on the primary outcome. Obtaining these data proved very difficult and as a result there was a large amount of missing or poor quality data, particularly in the GP arm. As a result of the study not recruiting to target and the high level of missing data in the GP arm, the results are reported descriptively without the planned statistical analysis being carried out.

In the DSN arm, 26 out of 29 (90%) participants were successfully contacted by a DSN within four weeks of the call-out (16/29 (55%) within two days and 25/29 (86%) within two weeks), and a discussion was held about their recent hypoglycaemic event (Figure 1). In the GP arm, 17 out of 26 (65%) had a telephone or face-to-face appointment with a healthcare professional in their practice within four weeks of the call-out. It is important to note that primary outcome data were only obtained for 26/46 (57%) of participants in this arm.

East Midlands Ambulance Service NHS Trust provided information on whether study participants called the ambulance service for an SH event on a second occasion during the following 24 hours, 72 hours and 12 months. Two out of 81 participants (2.5%) had a second call-out within 24 hours and 3/81 (3.7%) within 72 hours. Out of the 81 participants, 26 (32%) had a second call-out within 12 months (9% within 14 days, 11% within 30 days and 16% within 60 days). The second call-out rate was higher in the DSN arm compared to the GP arm (13 out of 35 (37%) and 13 out of 46 (28%) respectively). Data from East Midlands Ambulance Service NHS Trust central records showed that 2988 people in the two study sites called the ambulance service for an SH event during the 26-month study period and the second call-out rate within 12 months was 14.7%.

## Discussion

Despite the difficulties in recruitment, overall the study found that a successful telephone consultation was held with 90% of participants in the DSN arm compared to the GP arm (65%). Overall, 32% of participants called the emergency services for an SH event on a second occasion during the following 12 months and this rate was higher in the DSN arm than the GP arm (37% and 28% respectively). The second call-out rate in study participants was over twice the rate that occurred in the region as a whole during the study period (14.7%).

One of the primary objectives of a pathway of this design would be to reduce the number of repeat call-outs made by people with diabetes. One possible explanation for the repeat call-out rate in study participants being higher than occurred in the region as a whole is that taking part in the various research procedures, being given a booklet about the prevention of SH (an electronic version of which is freely available from the authors on request) and, in the majority of DSN participants, having a telephone consultation may have raised participants’ awareness and/or anxiety levels, encouraging them to call the emergency services when they had another SH episode. An alternative explanation could be that the 81 study participants were not representative of the 2988 people in the region who called out the ambulance service for an SH event during the study period. It is plausible that patients who experience more regular episodes of SH were likely to be referred to the study by virtue of increased interaction with ambulance staff members referring to the pathway. However, care needs to be taken when interpreting the different rates as the number of study participants was relatively small.

### Strengths

The study utilised a robust design and was successful in co-designing a care pathway with extremely good engagement and involvement from local ambulance staff. Despite this good engagement and uptake of training, recruitment rates were lower than anticipated. The study still provides useful insight into the difficulties of recruitment in emergency care settings and adds to the literature focusing on methodological considerations relating to studies of this type.

### Limitations

The main weakness of this study was the low recruitment, randomisation and consent rates resulting in recruitment being stopped when 54% of the target was reached. Previous research has reported on barriers to research in the pre-hospital sector, citing the workload imposed by the required research activities (e.g. checking eligibility, obtaining consent) on an already busy workforce as a major barrier to recruitment (Green et al., 2020; Pocock et al., 2019). Also, due to the pragmatic nature of the study and the number of trained ambulance staff needed, a maximum of only two hours’ training in the referral procedures was completed, with many staff completing a one-hour e-learning module. Data from telephone interviews with a number of the ambulance staff highlighting the barriers and facilitators to the successful implementation of study procedures will be published in a separate lessons learnt paper.

The chosen method of randomisation (sequentially numbered envelopes) was selected due to the need for it to be carried out quickly without contacting an external statistician; referral numbers in each arm differed slightly (73 and 89 referrals), however groups were well balanced in terms of age, sex and diabetes type.

The requirement of the research ethics committee to obtain consent in two stages resulted in loss of half the referrals who provided brief consent. A number of participants refused to give full consent, but in the majority of cases full consent was not obtained either because eligibility was not confirmed or because the participant could not be contacted using the contact information supplied (Figure 1). Obtaining primary outcome data for participants in the GP arm proved very difficult, resulting in a high level of missing or poor quality data which meant that the two arms could not be compared with confidence. The main problem was that a large number of practices had to be approached, most of which only had one patient in the study. Due to the low number of referrals to each practice over a relatively long time frame, the majority of practice staff were unfamiliar with the study, and the nature of information requested meant that it took some time to access medical records for each participant and extract all relevant data.

The rate of telephone consultations in the DSN arm was high, with 90% receiving a telephone call within four weeks, the majority (86%) being within two weeks, a rate which would be considered very successful if implemented in clinical care (Joint British Diabetes Societies for Inpatient Care, 2013). This is higher than occurred in the GP arm (65% within four weeks), although the amount of missing data is high. Comparison with the literature is difficult due to the heterogeneous nature and poor reporting of the pathways evaluated. An evaluation of the first 2000 referrals to a pathway delivered in the East of England between 2015 and 2016 (Sampson et al., 2017) reported comparable data showing that 72% had a face-to-face or telephone consultation with trained clinical educators following the call-out.

## Conclusion

In conclusion, our experiences highlight the difficulties of research in the pre-hospital sector, including ethical problems of gaining consent (Morrison et al., 2009) and staff training (Ankolekar et al., 2014; Armstrong et al., 2017). There was some evidence of higher rates of consultation in the DSN study arm, but the lack of power limits drawing conclusions about the significance of this. There is a need for future work to design and evaluate effective community referral pathways which improve diabetes management and minimise recurrence of SH, but an RCT with individual referral, 1:1 randomisation and a two-stage consent process has proved to be an infeasible study design.

## Acknowledgements

This research was supported by the National Institute for Health Research (NIHR) Collaborations for Leadership in Applied Health Research and Care East Midlands (CLAHRC-EM), the NIHR Leicester Biomedical Research Centre and the University of Leicester Centre for Black and Minority Ethnic Health. We would like to thank the EMAS ambulance staff who completed the training and referred patients to the study.

## Author contributions

KK,NS and MD conceived the idea. HD, AW, LG and CT designed and managed the study. JJ delivered the training. DS provided the data from EMAS. AW and HD wrote the main manuscript text. All authors reviewed the manuscript and approved the submitted version. AW acts as the guarantor for this article.

## Conflict of interest

KK has acted as consultant, advisory board member and speaker for Amgen, AstraZeneca, Bayer, NAPP, Lilly, Merck Sharp & Dohme, Novartis, Novo Nordisk, Roche, Berlin-Chemie AG/Menarini Group, Sanofi-Aventis, Servier and Boehringer Ingelheim. He has received EACME grants from Boehringer Ingelheim, AstraZeneca, Novartis, Novo Nordisk, Sanofi-Aventis, Lilly and Merck Sharp & Dohme.

MD has acted as consultant, advisory board member and speaker for Boehringer Ingelheim and Lilly, a consultant and speaker for Novo Nordisk, an advisory board member for Lexicon, an advisory board member and speaker for Sanofi and a speaker for NAPP and AstraZeneca. She has received grants in support of trials from AstraZeneca, Novo Nordisk, Sanofi-Aventis, Lilly, Boehringer Ingelheim and Janssen. All other authors have no competing interests to declare.

## Ethics

Ethics approval was granted by East Midlands – Nottingham 1 Research Ethics Committee (15/WM/0538 on 19 January 2016, and the study was prospectively registered (ISRCTN56314240)), the Health Research Authority and relevant NHS trusts.

## Funding

Funding for the study was provided by the National Institute for Health Research (NIHR), Collaborations for Leadership in Applied Health Research and Care East Midlands (CLAHRC-EM), now recommissioned as the NIHR Applied Research Collaboration East Midlands (ARC-EM). Funding was also obtained from Health Education East Midlands (HEEM) and East Midlands Academic Health Sciences Network (AHSN EM).
